# Molecular-genetic pathways of hepatitis C virus regulation of the expression of cellular factors PREB and PLA2G4C, which play an important role in virus replication

**DOI:** 10.18699/VJGB-23-90

**Published:** 2023-12

**Authors:** E.L. Mishchenko, A.A. Makarova, E.A. Antropova, A.S. Venzel, T.V. Ivanisenko, P.S. Demenkov, V.A. Ivanisenko

**Affiliations:** Institute of Cytology and Genetics of the Siberian Branch of the Russian Academy of Sciences, Novosibirsk, Russia Kurchatov Genomic Center of ICG SB RAS, Novosibirsk, Russia; Institute of Cytology and Genetics of the Siberian Branch of the Russian Academy of Sciences, Novosibirsk, Russia; Institute of Cytology and Genetics of the Siberian Branch of the Russian Academy of Sciences, Novosibirsk, Russia; Institute of Cytology and Genetics of the Siberian Branch of the Russian Academy of Sciences, Novosibirsk, Russia Kurchatov Genomic Center of ICG SB RAS, Novosibirsk, Russia; Institute of Cytology and Genetics of the Siberian Branch of the Russian Academy of Sciences, Novosibirsk, Russia Kurchatov Genomic Center of ICG SB RAS, Novosibirsk, Russia; Institute of Cytology and Genetics of the Siberian Branch of the Russian Academy of Sciences, Novosibirsk, Russia Kurchatov Genomic Center of ICG SB RAS, Novosibirsk, Russia Novosibirsk State University, Novosibirsk, Russia; Institute of Cytology and Genetics of the Siberian Branch of the Russian Academy of Sciences, Novosibirsk, Russia Kurchatov Genomic Center of ICG SB RAS, Novosibirsk, Russia Novosibirsk State University, Novosibirsk, Russia

**Keywords:** hepatitis C virus, HCV gene replication, replicase HCV, host factors, gene networks, phospholipase PLA2G4C, PREB protein, вирус гепатита С, репликация генома ВГС, репликаза ВГС, хозяйские факторы, генные сети, фосфолипаза PLA2G4C, белок PREB

## Abstract

The participants of Hepatitis C virus (HCV) replication are both viral and host proteins. Therapeutic approaches based on activity inhibition of viral non-structural proteins NS3, NS5A, and NS5B are undergoing clinical trials. However, rapid mutation processes in the viral genome and acquisition of drug resistance to the existing drugs remain the main obstacles to fighting HCV. Identifying the host factors, exploring their role in HCV RNA replication, and studying viral effects on their expression is essential for understanding the mechanisms of viral replication and developing novel, effective curative approaches. It is known that the host factors PREB (prolactin regulatory element binding) and PLA2G4C (cytosolic phospholipase A2 gamma) are important for the functioning of the viral replicase complex and the formation of the platforms of HCV genome replication. The expression of PREB and PLA2G4C was significantly elevated in the presence of the HCV genome. However, the mechanisms of its regulation by HCV remain unknown. In this paper, using a text-mining technology provided by ANDSystem, we reconstructed and analyzed gene networks describing regulatory effects on the expression of PREB and PLA2G4C by HCV proteins. On the basis of the gene network analysis performed, we put forward hypotheses about the modulation of the host factors functions resulting from protein-protein interaction with HCV proteins. Among the viral proteins, NS3 showed the greatest number of regulatory linkages. We assumed that NS3 could inhibit the function of host transcription factor (TF) NOTCH1 by protein-protein interaction, leading to upregulation of PREB and PLA2G4C. Analysis of the gene networks and data on differential gene expression in HCV-infected cells allowed us to hypothesize further how HCV could regulate the expression of TFs, the binding sites of which are localized within PREB and PLA2G4C gene regions. The results obtained can be used for planning studies of the molecular-genetic mechanisms of viral-host interaction and searching for potential targets for anti-HCV therapy.

## Abstract (ru)

В репликации генома вируса гепатита С (ВГС) участвуют как вирусные, так и хозяйские белки. Терапевтические подходы, основанные на подавлении активности неструктурных вирусных белков NS3, NS5A, NS5B, проходят клинические испытания разных уровней. Однако быстрые мутационные процессы вирусного генома и приобретение лекарственной устойчивости остаются одними из главных препятствий в борьбе с ВГС. Идентификация и исследование клеточных факторов, участвующих в репликации РНК ВГС, а также регуляция вирусом их экспрессии важны для понимания механизмов репликации вируса и разработки эффективных подходов противовирусной терапии. Известно, что белок PREB, связывающий регуляторный элемент пролактина, и цитозольная фосфолипаза А2 гамма (PLA2G4C) играют важную роль в формировании платформ репликации РНК ВГС, а также в функционировании вирусной репликазы. Экспрессия генов PREB и PLA2G4C значительно увеличена в присутствии ВГС, но механизмы ее регуляции вирусными белками до сих пор не изучены. В данной работе с применением технологии текст-майнинга, реализованной в программно-информационной системе ANDSystem, реконструированы генные сети регуляции экспрессии генов человека PREB и PLA2G4C белками ВГС. На основании анализа генных сетей мы выдвинули гипотезы о регуляторных эффектах белков ВГС на функции хозяйских факторов в результате белок-белковых взаимодействий. Среди вирусных белков наибольшее количество регуляторных связей выявлено у вирусной протеазы NS3. Предположительно NS3 в результате белок-белкового взаимодействия подавляет активность транскрипционного фактора NOTCH1, что обусловливает активацию экспрессии PREB и PLA2G4C. Анализ генных сетей и данных о дифференциальной экспрессии генов в присутствии ВГС позволил нам также выдвинуть гипотезы о регуляции вирусом экспрессии транскрипционных факторов, сайты связывания которых находятся в районах генов PREB и PLA2G4C, и действии этих транскрипционных факторов на регуляцию транскрипции PREB и PLA2G4C. Полученные результаты могут быть использованы при планировании исследований по изучению молекулярно-генетических механизмов взаимодействия вирус–хозяин и поиска потенциальных мишеней для разработки лекарств против ВГС.

## Introduction

The Hepatitis C virus (HCV) causes a dangerous liver disease, which, starting asymptomatic, turns into a chronic form and can lead to cirrhosis and hepatocellular carcinoma (Yamane et al., 2013). The HCV genome is represented by a plus-chain RNA (~9,600 nucleotides), encoding structural (Core, E1, E2) and non-structural (p7, NS2, NS3, NS4A, NS4B, NS5A, NS5B) proteins. It also contains 5ʹ- and 3ʹ-untranslated regions (UTR) necessary for translating the viral polyprotein and replicating the viral genome (Bartenschlager et al., 2013). Structural glycoproteins E1 and E2 are localized on the viral bilayer lipid envelope surrounding the nucleocapsid, which consists of multiple copies of the Core protein and RNA genome. The p7 protein has membrane cation channel properties; proteins NS2 and NS3/NS4A are proteases that process the viral polyprotein. NS3 also has helicase activity; NS4B and NS5A can modify endoplasmic reticulum (ER) membranes to form vesicular membrane structures – platforms for the replication of the HCV genome. NS5B is an RNA-dependent RNA polymerase. The complex of non-structural proteins NS3–NS5B, which also involves host factors, performs the function of viral replicase in the host cell (Moradpour et al., 2007). The virus genome is highly heterogeneous due to the high error rate of the RNA-dependent RNA polymerase NS5B. This property of NS5B is considered the main reason for the virus’s rapid acquisition of drug resistance (Powdrill et al., 2011).

Currently, a great deal of research is directed towards identifying and studying the properties of cellular factors involved in modifying ERmembranes to form vesicle clusters in which the HCV RNA genome replicates, which are part of the viral replicase. For instance, it has been established that the receptor for activated C kinase 1 (RACK1) associates with NS5A and the ATG14L-Beclin1-Vps34-Vps15 autophagosome formation initiation complex, stimulating the formation of vesicular membrane structures (Lee et al., 2019). The early endosome (EE) protein Rab5, regulating endocytosis and EE fusion, and the late endosome (LE) protein Rab7, enhancing LE transport to lysosomes, are associated with NS4B and involved in the biogenesis of these membrane structures (Manna et al., 2010). The small GTPase Rab18∙GTP on lipid droplet (LD) membranes interacts with the viral protein NS5A on the ER membrane. The association of LD and ER membranes due to the direct interaction of Rab18∙GTP and NS5A leads to the localization of HCV replicase complexes near LDs and stimulates HCV RNA replication (Salloum et al., 2013).

Phosphatidylinositol 4-kinase IIIα (PI4KIIIα) is important in forming membrane vesicular structures and replication complexes. Through protein-protein interaction, NS5A stimulates the activity of PI4KIIIα, leading to the formation of phosphatidylinositol-4-phosphate (PI4P), which recruits and coordinates viral and host proteins on the membrane that contains PI4P-affine lipid-binding domains (Berger et al., 2011; Reiss et al., 2011). Moreover, HCV can regulate the expression of cellular factors that play an important role in virus replication. For example, cytosolic phospholipase A2 gamma (PLA2G4C), which hydrolyzes membrane phosphoglycerides to form free fatty acids and lysophosphatidate and directly affects the structure, shape, merger, and interaction of the membranes with proteins (Brown et al., 2003), has several times increased expression at both RNA and protein levels in the presence of HCV RNA (Xu et al., 2012).

The expression of the PREB gene (prolactin regulatory element binding protein) is also significantly increased in the presence of HCV (Kong et al., 2016). The PREB protein functions as a regulatory factor for COPII vesicle budding from the ER membrane (LaPointe et al., 2004), associates with NS4B, is involved in the formation of membrane vesicular structures and is localized in the active HCV replication complex through interaction with NS4B (Kong et al., 2016). Despite accumulated evidence of increased PREB and PLA2G4C expression in the presence of HCV, the molecular mechanisms regulating the expression of these host factors are poorly understood.

The technology of text mining is a useful tool for studying molecular-genetic interactions. We previously developed the software and information system ANDSystem (IvanisenkoV.A. et al., 2015, 2019; Ivanisenko T.V. et al., 2020, 2022), which implements a full cycle of knowledge engineering, including automatic extraction of information from scientific publications and factographic databases, integration, and representation of information in the form of semantic networks in the knowledge base, as well as providing user access to the knowledge base for the reconstruction and analysis of gene networks. ANDSystem was used to solve a wide range of tasks, including analyzing the interactome of Hepatitis C virus proteins with human proteins, interpreting metabolomic analysis results, gene prioritization tasks, searching for new potential drug targets, and others. In particular, the analysis of proteinprotein interactions of HCV and human proteins allowed us to reconstruct potential pathways of regulating the external pathway of apoptosis by viral proteins (Saik et al., 2016), as well as to study the features of HCV protein regulation of genes prone to aberrant methylation in hepatocellular carcinoma (Antropova et al., 2022). Based on the data of metabolomic analysis of the blood plasma of patients with COVID-19, regulatory pathways describing the control of human metabolic pathways by SARS-Cov-2 proteins were reconstructed, and it was shown that a number of non-structural viral proteins had the most significant regulatory impact (Ivanisenko V.A. et al., 2022). With the help of reconstruction and analysis of gene networks, new methods of gene prioritization were proposed, which were used to search for candidate genes associated with lymphedema as well as with major depressive disorder (Yankina et al., 2018; Saik et al., 2019). Using ANDSystem, new potential pharmacological targets for treating comorbid conditions of asthma and hypertension were proposed (Saik et al., 2018a, b).

In our work, using the ANDSystem software information system, we reconstructed and analyzed the pathways of HCV protein regulation of the expression of cellular factor genes PLA2G4C and PREB, which play an important role in the formation of membrane vesicular structures – the platform for viral RNA replication, and in the functioning of the viral replicase. Through computer analysis, 28 human transcription factors (TFs) under the control of HCV were found which could participate in the regulation of PLA2G4C and PREB expression. It turned out that out of these TFs, 16 proteins participate in the regulation of PLA2G4C, 23 – in the regulation of PREB, and 11 are common. Based on the analysis of gene networks and data on differential gene expression, hypotheses have been put forward about the regulatory effects of viral proteins on the functions of TFs with which they form complexes as a result of protein-protein interactions, as well as the regulatory effects of these TFs on the expression of PLA2G4C and PREB.

## Materials and methods

Obtaining the list of differentially expressed genes (DEGs) of human proteins in the presence of HCV proteins. Using RNA sequencing results available at the NCBI GEO resource (http://www.ncbi.nlm.nih.gov/geo) (Edgar et al., 2002), a list of human genes differentially expressed in Huh7.5.1 hepatocytes under HCV infection conditions was obtained via the GSE66842 identifier. The RNA sequencing results were analyzed using the GEO2R tool, allowing to obtain statistical processing results and data visualization on differential gene expression under experimental conditions. We selected statistically significant DEGs at the control point “10 days after HCV infection” (GSE66842). The study also used transcriptome analysis results of differential gene expression in Huh.7.5 hepatocytes at the control point “72 hours after HCV infection” (Papic et al., 2012). These results were combined into a final list of DEGs to reconstruct gene networks.

Identification of transcription factors. Transcription factors, the binding sites of which are located in the PREB and PLA2G4C genes, as well as in flanking regions of these genes within a range of ±2,000 bp, were extracted from the GTRD database (http://gtrd20-06.biouml.org/) (Yevshin et al., 2017; Kolmykov et al., 2021), which integrates studies on genome organization. For gene network construction, the TF genes differentially expressed under Hepatitis C virus infection conditions were selected

Reconstruction and analysis of molecular genetic pathways of PREB and PLA2G4C gene expression regulation by HCV proteins using ANDSystem. Molecular genetic pathways for regulating host factors PREB and PLA2G4C expression by HCV proteins were reconstructed using ANDSystem and its graphical user interface ANDVisio. The ANDVisio program accesses the ANDSystem knowledge base, which contains over 40 million facts about intermolecular interactions, including protein-protein interactions, gene expression regulation, activity regulation, degradation, and protein transport

**Fig. 1.**
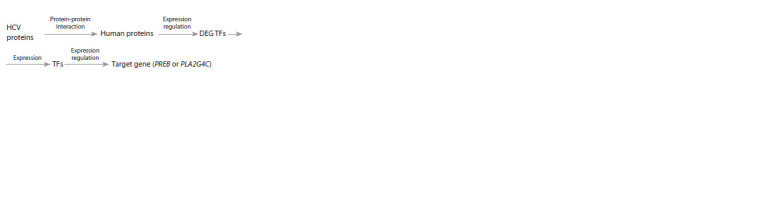
Scheme for constructing regulatory molecular-genetic pathways for modulating the expression of host factor genes by HCV proteins.

The construction of regulatory molecular genetic pathways describing interactions between HCV proteins and human proteins and genes was carried out using the “Pathway Master” module of the ANDVisio program. The relationships between the participants of these pathways, including protein-protein interactions and gene expression regulation, are arranged according to the scheme (Fig. 1).

## Results and discussion

Reconstruction of the interactome of human proteins and HCV proteins

**Fig. 2.**
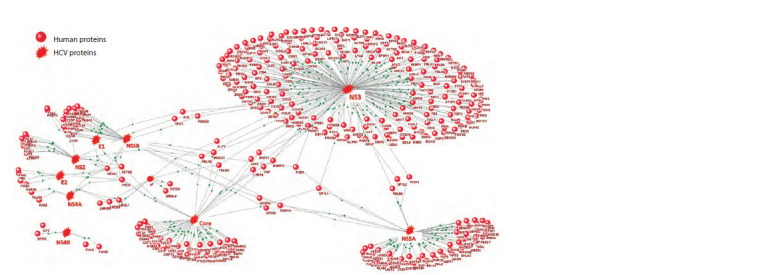
Graph of interactions between human proteins and HCV proteins, reconstructed using the ANDSystem software and information system. Black lines indicate protein-protein interactions.

Using the ANDSystem software and information system, an interactome of 10 HCV proteins with 333 human proteins was reconstructed (Fig. 2). It turned out that 195 human proteins interact with NS3, 59 – with NS5A, 50 – with Core, 26 – with NS5B, 15 – with NS2, 7 – with E2 and p7, 6 – with NS4A, 5 – with E1, 4 proteins – with NS4B. The gene network illustrates that only a few human proteins interact with more than one HCV protein. Among them are transcription factors potentially regulating the expression of target genes PREB and PLA2G4C.

Reconstruction of molecular-genetic pathways regulating the expression of PREB and PLA2G4C genes by HCV proteins

Published scientific results indicate that the expression of cellular factors PLA2G4C (Xu et al., 2012) and PREB (Kong et al., 2016) is significantly enhanced in the presence of HCV proteins. These host factors play an important role in HCV replication. They are involved in forming membranous vesicular structures – compartments of viral RNA replication, and in the functioning of the HCV replicase complex (Xu et al., 2012; Kong et al., 2016). However, the molecular-genetic mechanisms for increasing the expression of PREB and PLA2G4C in the context of HCV infection have not been studied to date. Transcription factors (TFs) regulated by viral proteins were identified using information on differential gene expression. It should be noted that in our study, we did not consider TFs, the expression of which did not change under conditions of HCV infection. The GTRD database extracted lists containing 432 and 693 TFs, the binding sites of which are in the regions of PREB and PLA2G4C genes, respectively. Among many transcription factors, 92 TFs were selected, the genes of which are differentially expressed in the presence of HCV proteins (69 and 63 TF genes for PREB and PLA2G4C, respectively, and 40 TFs common for both target genes).

**Fig.3.**
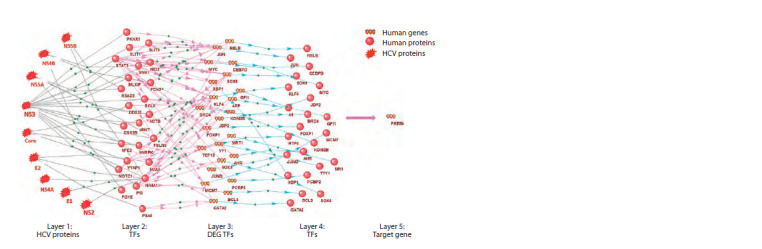
Gene network of molecular-genetic pathways regulating the expression of the PREB gene in conditions of HCV infection Here and in Fig. 4: black lines – protein-protein interactions; pink arrows – expression regulation; blue arrows – expression.

**Fig. 4.**
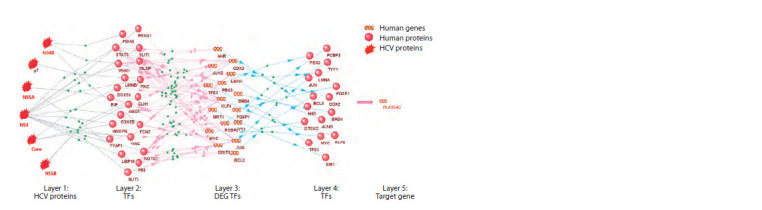
Gene network of molecular-genetic pathways regulating the expression of the PLA2G4C gene under HCV infection conditions.

Using ANDSystem, the molecular-genetic pathways regulating the expression of PREB and PLA2G4C by HCV proteins were reconstructed and analyzed (Figs. 3 and 4). Among the regulatory pathways, the first layer of which were HCV proteins, and the final ones were PREB and PLA2G4C genes, there turned out to be 28 out of 92 TFs, indicating the regulation of these TFs by viral proteins.

Figure 3 illustrates the regulatory molecular-genetic pathways of PREB expression by HCV proteins. These pathways include 24 proteins presented in layer 2, 23 participants in layer 4, and their encoding genes in layer 3. As shown in the gene network graph, only 23 out of 69 TFs were included in the regulatory pathways, suggesting that these specific TFs may regulate the transcription of the PREB gene under HCV infection conditions.

The gene network in Figure 4 illustrates the pathways of PLA2G4C expression regulation by HCV proteins. In the GTRD database, 63 TF binding sites were found in the regulatory regions of the PLA2G4C gene, which are differentially expressed genes (DEGs). Only 16 out of these 63 TFs were part of the regulatory pathways. This suggests that these particular TFs presumably regulate the transcription of the PLA2G4C gene under HCV infection conditions. It was previously shown that the NS3 protein of the Hepatitis C virus stimulates the activity of the TF STAT3 (Machida et al., 2006). Moreover, STAT3 significantly enhances the transcription of the MYC gene (Kiuchi et al., 1999; Papic et al., 2012). Furthermore, it was demonstrated in a study (Xiong et al., 2017) that the alteration of MYC expression enhanced PLA2G4C expression, which aligns with the regulatory pathway we identified. Similarly, the positive regulation of XBP1 expression by STAT3 (Diehl et al., 2008) and the increased expression of XBP1 (Papic et al., 2012) in the presence of HCV may account for the activating effect of XBP1 on PLA2G4C transcription.

The use of ANDSystem allowed us to propose hypotheses about the regulation of TF expression by HCV proteins interacting with the regulatory region sites of the PREB and PLA2G4C genes (see Figs. 3 and 4). It should be noted that 11 TFs were simultaneously represented among the regulators of PREB and PLA2G4C. Based on the data on differential gene expression and the nature of regulatory molecular-genetic pathway connections, we can hypothesize about the effect these TFs (layer 4) have on the transcription of PREB and PLA2G4C (see the Table). For example, the increased expression of the layer 3 TF gene and positive regulation by the layer 2 TF may lead to the activation of PREB and PLA2G4C transcription. Specifically, from the regulatory pathways, it follows that the TF CEBPD positively regulates the expression of PREB, as the expression of CEBPD is positively regulated by STAT3 (layer 2) and is elevated in the presence of HCV (Papic et al., 2012). Conversely, the reduced expression of the layer 4 TF in the presence of HCV and the negative sign of expression regulation between layer 2 and 3 participants explain the inhibitory effect of the TF on the transcription of PREB and PLA2G4C.

**Table 1.**
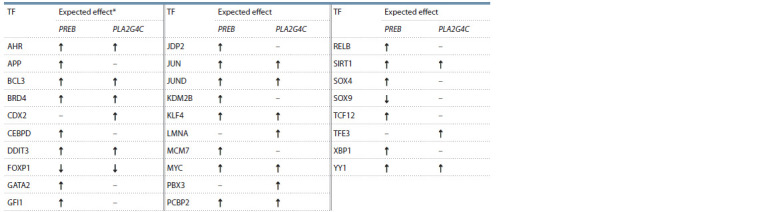
The expected effect of layer 4 TFs on the expression of PREB and PLA2G4C * «↑» – positive regulation, «↓» – negative regulation, «–» – no regulation.

The studies show that the Core HCV protein increases the expression of NR4A1 (Tan, Li, 2015), while the transcription factor NR4A1 inhibits the expression of the SOX9 gene (Hu et al., 2014). In the regulatory pathways we reconstructed, NR4A1 is a transcription factor of layer 2, interacts with six HCV proteins (Core, E1, E2, NS2, NS4A, NS5B), and has a negative effect on SOX9. Therefore, the transcription factor SOX9, inhibited at the RNA level under HCV infection conditions, presumably reduces the expression of the PREB gene. The hypotheses we proposed based on gene network analysis should be experimentally confirmed in the future

Analyzing the reconstructed gene networks allowed us to propose hypotheses about how viral proteins might affect the function of TFs with which they form complexes due to protein-protein interactions. These hypotheses were based on the structure of regulatory molecular-genetic pathways and data on differential gene expression, similar to the hypotheses about regulating PREB and PLA2G4C by TFs. A viral protein has a negative effect on the function of a protein from layer 2 of the regulatory pathway as a result of physical interaction with it in the following cases: (1) the layer 2 participant is connected to a participant from layer 3 by positive regulation of expression type, and the expression of the layer 3 participant is reduced in the presence of HCV; (2) the layer 2 participant is connected to a participant from layer 3 by negative regulation of expression type, and the expression of the layer 3 participant is increased in the presence of HCV. A viral protein has a positive effect on the function of a protein from layer 2 in the following cases: (1) the layer 2 participant is connected to a participant from layer 3 by positive regulation of expression type, and the expression of the layer 3 participant is increased in the presence of HCV; (2) the layer 2 participant is connected to a participant from layer 3 by negative regulation of expression type, and the expression of the layer 3 participant is reduced in the presence of HCV.

According to the reconstructed regulatory molecular-genetic pathways, the largest number of regulatory connections among the HCV proteins was identified for the viral protease NS3. One of the proteins directly interacting with NS3 is the TF NOTCH1. Numerous scientific studies of this TF have been published; however, we did not find information about the effect of NS3 on the function of NOTCH1 due to proteinprotein interactions. From analyzing regulatory pathways and differential gene expression data, we hypothesized that NS3 suppresses NOTCH1 activity due to protein-protein interaction. It was previously shown that NOTCH1 activates the transcription of SOX9 (Zong et al., 2009) and inhibits KLF4 (Xue et al., 2016), which would lead to a negative effect on the transcription of PREB and PLA2G4C. However, the actual change in the expression of target genes and their TFs SOX9 and KLF4 aligns with the hypothesis about the suppression of NOTCH1 activity by the viral protein NS3.

## Conclusion

Using the ANDSystem software system, molecular-genetic pathways of regulation of PLA2G4C and PREB gene expression by HepatitisC virus proteins have been reconstructed and analyzed. The protein products of these genes are essential for HCV replication, as they participate in the modification of membranes with the formation of membrane vesicle clusters, which are compartments of HCV genome replication and are also involved in the composition and functioning of the HCV replicase. The theoretical data obtained in our work can be useful for planning studies on the mechanisms by which HCV uses human proteins for its genome replication and for searching for potential targets for antiviral therapy.

## Conflict of interest

The authors declare no conflict of interest.
